# Color stability of fruit yogurt during storage

**DOI:** 10.1007/s13197-019-03668-y

**Published:** 2019-03-19

**Authors:** Iwona Ścibisz, Małgorzata Ziarno, Marta Mitek

**Affiliations:** 1 Division of Fruit and Vegetables Technology, Faculty of Food Sciences, Warsaw University of Life Sciences (SGGW-WULS), Nowoursynowska St. 166, 02-787 Warsaw, Poland; 2 Division of Milk Biotechnology, Faculty of Food Sciences, Warsaw University of Life Sciences (SGGW-WULS), Nowoursynowska St. 166, 02-787 Warsaw, Poland

**Keywords:** Strawberry, Sour cherry, Blueberry, Yogurt, Color, Anthocyanin

## Abstract

This article describes the evaluation of the anthocyanins stability in yogurts with strawberry, sour cherry, and blueberry fruit preparation during 8-week storage period under refrigerated condition. The differences in anthocyanin degradation rate and color changes between stirred yogurts and fruit-on-the-bottom yogurts (fruit preparation was on-the-bottom of package) were compared. Anthocyanin content in fruit yogurts showed a significant decreased during the storage, especially for the first 2 weeks. There were differences in the rate of pigment degradation between yogurt obtained from a different species of fruit. The half-life of the pigments in stirred yogurt with the preparation of strawberry, sour cherry, and blueberry was found to be 5.5, 6.7, and 19.0 weeks, respectively. The addition of fruit preparation on the bottom of yogurt could be used to reduce the pigment degradation during storage. The half-life of anthocyanin in fruit-on-the-bottom yogurts was 39–63% higher than in the blending samples. A significant alteration in the pigment profile during storage of blueberry yoghurt was observed. The proportion of malvidin-glucosides and acylated anthocyanins increased with time of storage, with a concomitant decrease in proportion of petunidin, delphinidin and peonidin derivatives.

## Introduction

Yogurt is one of the most popular fermented dairy products all over the world. Recently, fermented milk products have been gaining more attention by consumers, both because of their nutritional benefits, and the presence of ingested live microorganisms. The addition of fruit preparation, rich in natural antioxidants and fibers, may also enhance the possible health effects of yogurt products (Şengül et al. 2012).

Color is an important factor of fruit yogurt quality, influencing consumer`s acceptability of the product. It is one of the first characteristics perceived by the senses and is used by consumers for the evaluation of the quality of food products (Giusti and Wrolstad 2003). Unfortunately, the attractive color of the fruit yogurt does not prevail through storage. The shelf life of yogurt products is only 6–8 weeks under refrigerated condition, depending on the production method and packaging material. The addition of fruit preparations in the production of yogurt is a potential source of yeast and mold contamination. If fruit preparations are heated and post-processing contamination is avoided, the shelf-life of the fruit-flavored yogurts can also be extended to 8 weeks (MacBean 2009). However, in this short-time period, the shade of yogurt color may become lighter, unattractive for consumers. The dosage of fruit preparation in dairy products range only between 5 and 25%, and therefore obtaining the appropriate color of the products is a very demanding challenge (Fügel et al. 2005). The food industry has used natural food colorings by the application of intensively pigmented materials to improve the color and acceptation of fruit yogurts during its shelf life. However, the addition of colorants is not well accepted by consumers, who believe that the food producer hides the poor quality of food in this way (McAvoy, 2014).

A red and blue–purple colors of fruit yogurt depends on the concentration of anthocyanins. Anthocyanins are water-soluble plant pigments, which belong to the flavonoid class of compounds. The color expression is highly influenced by the molecular structure of pigments. Acylation of molecule and increased in glycosidic substitution are reported to improve the stability of these pigments. As the methoxylation of aglycone degree increases, it observes the shift in hue of anthocyanin solution from orange to purple (Heredia et al. 1998). pH of food matrix is another factor that influences the stability and color of the pigments. Cabrita et al. (2000) observed in a model system that anthocyanidin 3-glucosides showed higher color intensity and higher stability at pH 2.4–3.2 than at pH 4–5 during storage at 10 °C. In buffered aqueous solutions at pH 2.4 and 3.2, 82% and 89% of cyanidin-3-glucoside, respectively, was still intact after 60 days of storage, while at pH 5.0, cyanidin-3-glucoside was completely degraded within 15 days of storage.

Besides their colorant properties, anthocyanins display a wide range of biological activity including antimicrobial, antimutagenic, anticancer, antitumor and antioxidant activity. The ability of anthocyanins to prevent of cardiovascular disease, hepatic damage, and neurodegenerative processes was also confirmed (Pascual-Teresa and Sanchez-Ballesta 2008).

Studies showed that the stability of anthocyanins during the storage is affected by temperature, pH value, water activity, light exposure, and microbial activity. The presence of enzymes, oxygen, metallic ions, sulfur dioxide, and ascorbic acid can also influence pigment degradation. The improvement of anthocyanin stability has been attributed to intermolecular copigmentation among anthocyanins and other phenolic compounds and self-association between anthocyanins themselves (Pascual-Teresa and Sanchez-Ballesta 2008; Patras et al. 2010). Moreover, Baiano et al. (2018) showed that the stability of delphinidin-3-O-glucoside and malvidin-3-O-glucoside during the simulation of wine aging depended on the yeast strains. However, published data mostly concern on monitoring the changes of anthocyanin content in acidic food products (pH < 3.5) like juice, wine, purees, pulp, jam, or dried fruit (Baiano et al. 2018; Cao et al. 2012; Ertan et al. 2018; Picariello et al. 2017). A little attention has so far been paid to the fermented milk products with addition of fruit preparation. Investigations comparing the factors influencing anthocyanin stability in yogurt matrix are scarce in the literature. The degradation of anthocyanins during the storage was detected in fruit yogurt products such as grape yogurt (Karaaslan et al. 2011), purple carrot and *Berberis boliviana* L. yogurts (Wallace and Giusti 2008) and strawberry yogurt (Oliveira et al. 2015). Researches have indicated that the stability of anthocyanins depends on fat content in yogurt and the degree of pectin methoxylation used in yogurt production. Adding anthocyanin extract before fermentation of milk also could influence the pigment content in yogurt (Sun-Waterhouse et al. 2013; Wallace and Giusti 2008).

In this study, we assessed the impact of two industry practices (addition of the fruit preparation to the mass of yogurt or on-the-bottom of package of yogurt) on the anthocyanin loss and color changes during the storage. We have determined anthocyanin stability of strawberry, sour cherry, and blueberry yogurts. Our results could be useful for the development of an effective strategy for the production of dairy food with improved color stability.

## Materials and methods

### Materials, chemicals and solvents

Commercial UHT milk was obtained from Mlekpol Dairy Co. (Grajewo, Poland). The culture of YC-X16 (*Streptococcus thermophilus* and *Lactobacillus delbrueckii* subsp. *bulgaricus),* was purchased from Chr. Hansen (Hørsholm, Denmark). A commercial preparation of low-methoxyl pectin (NEC A2) was purchased from Pektowin (Jasło, Poland) and tapioca native starch Novation®3600 was purchased from National Starch & Chemical GmbH (Hamburg, Germany). The anthocyanin standards were purchased from LGC Standards (Teddington, UK) and from Extrasynthese (Lyon, France). Others chemicals used were of analytical grade or HPLC gradient grade and were purchased from Avantor Performance Materials (Gliwice, Poland) and from Sigma-Aldrich (St. Louis, MO, USA).

### Plant material

About 6 kg of strawberry (var. Senga-Sengana) and highbush blueberry (var. Patriot) and 7 kg of sour cherry (var. Kelleris) were used for fruit preparation making. Fruits were obtained from local growers (strawberry, blueberry) and from the experimental orchard of Faculty of Horticulture, Biotechnology and Landscape Architecture SGGW-WULS (sour cherry), located near Warsaw (Poland). After harvest, fruits were immediately packed in polyethylene bags, frozen and kept at − 25°C for 2 months for further production of yogurt fruit preparation.

### Fruit preparation processing

Production of fruit preparation was performed on a laboratory scale. The frozen blueberry, strawberry, and sour cherry were kept at room temperature to thaw for 1–2 h. Semi-thawed fruit of sour cherry was mechanically pitted using pitting machine with needles pushing each pits out of cherry. Unfrozen fruit, sugar, starch, and the preparation of low-methoxyl pectin were processed under vacuum conditions (60°C) in order to obtain a yogurt fruit preparation containing 600 g fruit per kg and the final sucrose concentration of 40°Brix. Pectin and starch dosage (6 g/kg and 4 g/kg, respectively) was set according to the supplier’s recommendations for mass production.

Fruit, crystallized saccharose, and water were gently mixed together. The mixture was allowed to boil at 60 °C for 20 min before the pectin solution and starch were added. A commercial pectin and starch were dissolved in 100 mL of hot water with the aid of a mixer. The temperature was raised to 60°C and held there for 10 min. Fruit preparation was hot-packed to glass jars (330 mL). The jars were then sealed and pasteurized at 90 °C for 10 min in boiling water bath. After pasteurization, the jars were cooled to 30 °C using chilled water. The yogurt fruit preparation was done in duplicates. When samples had cooled to room temperature, they were incorporated into yogurts.

### Yogurt processing

The white mass of yogurt was prepared using commercial UHT milk and non-fat milk powder, which was added to increase the milk solid content. Milk was inoculated with starter culture containing *Streptococcus thermophilus* and *Lactobacillus delbrueckii* subsp. *bulgaricus*. Yogurt was fermented in a 37 °C water bath about 4.5 h until pH was reduced to 4.7 and then rapidly cooled to 14 °C. Afterwards the fruit preparation was incorporated into the obtained yogurt sample.

### Production of fruit yogurt

Two types of strawberry, sour cherry, and blueberry yogurts were prepared with the addition of 20% of fruit preparation in sterile glass jars with screw caps. The blending yogurt was made by adding the fruit preparation to the fermented mass of yogurt and mixed using mechanical agitator. Fruit pieces were designed to be interspersed throughout the yogurt. In the fruit-on-the-bottom yogurts, fruit preparations were added at the bottom of jars while yogurt was being deposited on the top of package. The fruit yogurts were stored in the dark at 5 ± 1°C. During this time, samples at 1, 2, 4, 6, and 8 weeks of storage were taken.

### Analytical methods

The physicochemical analysis and microbial population were determined directly after the production of fruit yogurt and after 8 weeks of storage. The pH was measured with a digital pH-meter at 20 °C. Total acidity was determined by potentiometric titration with NaOH 0.1 N to pH 8.2. Titratable acidity was expressed as the percentage of lactic acid on a 100 g of yogurt. Lactic acid content was determined by high-performance liquid chromatography using a Shimadzu instrument (LC-10AT) comprising a quaternary solvent pumping system (FCV-10ALVP), degasser model DG-4400, detector UV–Vis (SPD-10AVP), column oven (CTO-10ASVP), autosampler (SIL-20AHT) and LCsolution data collection software. The extraction was performed according to Adhikari et al. (2002). A Cosmosil 5C18-PAQ (4.6 mm × 150 mm) column was used. The elution was performed using 20 mmol phosphoric acid at a flow rate of 1 mL/min. Lactic acid was detected at 210 nm and identified according to retention time by comparing with the standard. The Kjeldahl method was used to determine the total protein content in fruit yogurt (AOAC 1995), fat content was measured by the Gerber methods (Wehr 2004). Total phenolic compounds were determined by the Folin–Ciocalteau method (Gao et al. 2000). The calibration curve was performed with gallic acid and the results were expressed as mg of gallic acid equivalents per 100 g of yogurt (mg GAE/100 g).

An HPLC system and ZIC®-HILIC column (4.6 mm × 150 mm, 5μm, SeQuant, Sweden) was used to measure the concentration of l-ascorbic acid in yogurt according to the method of Divelos et al. (2010) with some modifications. Homogenize sample (2–5 g) was extracted in 40 mL of 10 mM oxalic acid. The extract was centrifuged at 3200×*g* for 10 min at 4 °C and the supernatant was diluted 2 times of acetonitrile: water with 66.7 mM ammonium acetate (850:150 v/v) solution and filtered on a PTFE 0.45 µm filter prior to the analysis. The chromatographic analyzes were carried out using acetonitrile solution as a mobile phase. The flow rate was 0.5 mL/min and the absorbance at 240 nm was monitored. The calibration curve was constructed using freshly prepared l-ascorbic acid solution (in the range of 1–50 µg).

Determination of the population of *L. delbrueckii* subsp. *bulgaricus* and *S. thermophilus* cells was carried out with the traditional plate method using MRS and M17 media, respectively (ISO 7889, 2003). The plates were incubated under anaerobic (*L. delbrueckii* subsp. *bulgaricus*) and aerobic *(S. thermophilus)* conditions for 72 h at 37 °C, after which colonies were counted.

Anthocyanins were determined by Shimadzu HPLC. The extraction of anthocyanins from 40 g samples of homogenized fruit yogurts was conducted four times, with a 50 mL portion of the solution containing methanol/water/hydrochloric acid (700/300/1, v/v/v). The extracts were retained at − 23 °C for 1 h to allow protein precipitation. The sample was centrifuged at 6880×*g* for 10 min and the supernatant was collected. The sample was rotary evaporated to complete the evaporation of methanol and brought to the volume of 25–100 mL using an aqueous phosphoric acid solution (1.0 g/L). About 10 mL of the anthocyanin extract was injected into a preconditioned Sep-Pak C_18_ cartridge. Samples were filtered through PTFE 0.45 μm filters before HPLC analyses.

Anthocyanins were separated on a Luna C18(2) RP (5 μm) 250 × 4.6 column (Phenomenex) at 25 °C. Anthocyanins from blueberry yogurt were eluted with a gradient of 10% acetic acid (mobile phase A) and acetonitrile (mobile phase B) starting with 6% phase B, then 9% B for 7 min, 11% B for 11 min, 14% B for 3 min, 22% B for 5 min, 30% B for 4 min, 6% B for 10 min, used at the flow rate of 1 mL/min. For the separation of anthocyanins in strawberry and sour cherry, the mobile phases were (A) formic acid/water (100:900, v/v) and (B) acetonitrile/formic acid (900:100, v/v). The gradient elution was as follows: 0–2 min, 2% B; 2–25 min, 2–20% B; 25–30 min, 20–40% B; 30–34 min, 40–2% B; 34–38 min, 2% B. Monomeric anthocyanins in sour cherry and strawberry yogurts were identified by comparing their retention time, elution order, and UV spectra to those of authentic standards or to previously reported data. Because the identification of blueberry anthocyanins is quite complicated due to the presence of large number of compounds, the structural identification of blueberry anthocyanins was also confirmed by MS analysis. Anthocyanins isolated from blueberry yogurt were analyzed with MS detection, using a Shimadzu single quadrupole mass spectrometer (LCMS 2010), column and condition described above. Electrospray ionization was performed in a positive ion mode. Mass spectra were measured in the range of 400–600 m/z. Different monomeric anthocyanins were quantified by comparing their peak areas in the chromatograms at 520 nm with the peak area of cyanidin-3-glucoside.

Color was measured on yogurts using a Konica Minolta colorimeter model CM-3600d having an 8 mm diameter viewing area, with the standard observer (10-degree visual field) and illuminant D65. The instrument was calibrated with a standard white and black reference tiles. A sample of fruit yogurt was homogenized and placed in a 2-cm-thick glass cuvette.

### Kinetics of anthocyanin degradation and statistical analysis

The first-order kinetic model was used to describe the degradation of anthocyanins during the storage of yogurts. The reaction rate constant (*k*) and life-time (*T*_1/2_), defining as the time in which half of the anthocyanin content decays, were calculated by the following equations:

1 $$ C = C_{o} e^{ - kt} $$

2 $$ T_{1/2} = \left( {\ln 2} \right)/k $$

where *C*_*o*_ is the initial concentration of anthocyanins; *C* is the anthocyanin concentration after *t* days of storage, *t* is the storage time (weeks), *k* is the reaction constant (weeks^−1^).

The data obtained were analyzed statistically using Statgraphics Centurion XVI software program. The means of physicochemical analysis and viable microbial counts of yogurts were analyzed by one-way analysis of variance, and the results were compared by Tukey test. For monomeric anthocyanin content and color of storage yogurt, the two-way analysis of variance was performed to evaluated the significant effect of two factors: the storage time and the method of fruit preparation addition. Differences between samples were considered to be statistically significant when *p* < 0.05.

## Results and discussion

### The changes of the physicochemical composition and viable microbial counts during storage of fruit yogurts

The physicochemical composition of the yogurts is shown in Table 1. Strawberry yogurts directly after the production presented a higher pH and lower acidity than sour cherries and blueberry yogurts, while all products contained similar amounts of lactic acid and this was due to the difference in the acidity of fruit preparation incorporated into yogurts. The mean of titratable acidity of all yogurts stored 8 weeks was significantly higher than that of the yogurts directly after the production. These results suggest that the production of organic acid is continuing at refrigeration temperatures, and this may affect the stability of color and sensory characteristics of yogurts. The concentration of lactic acid in stored products ranged from 0.56 g/100 g in blueberry blending yogurt to 0.71 g/100 g in strawberry fruit-on-the-bottom yogurt. During the storage, major changes were observed in the acidity of yogurts compared to pH. The presence of exogenous buffer constituents in yogurt such as proteins, citrates, phosphates, lactates can caused that the variations in pH are less pronounced than the variations in acidity (Tamime and Deeth 1980).

**Table 1:** Physicochemical composition and viable microbial counts of fruit yogurts directly after production and after 8 weeks of storage

Parameters	Type of yogurt	Storage time (weeks)	Fruit preparation		
			Strawberry	Sour cherry	Blueberry
pH	Blending	0	4.15^a^	4.01^a^	4.07^a^
		8	4.08^ab^	3.96^ab^	3.99^b^
	Fruit-on-the-bottom	0	4.15^a^	4.03^a^	4.09^a^
		8	4.05^b^	3.94^b^	3.97^b^
Titratable acidity (%)	Blending	0	0.93^b^	1.06^b^	1.03^b^
		8	1.02^a^	1.13^a^	1.08^a^
	Fruit-on-the-bottom	0	0.92^b^	1.04^b^	1.01^b^
		8	1.06^a^	1.16^a^	1.10^a^
Lactic acid content (g/100 g)	Blending	0	0.52^c^	0.51^c^	0.52^c^
		8	0.62^b^	0.58^b^	0.56^b^
	Fruit-on-the-bottom	0	0.54^c^	0.53^c^	0.53^c^
		8	0.71^a^	0.68^a^	0.63^a^
Protein content (%)	Blending	0	3.09^a^	3.04^a^	2.99^a^
		8	3.03^a^	3.03^a^	3.02^a^
	Fruit-on-the-bottom	0	3.02^a^	3.00^a^	3.01^a^
		8	3.01^a^	3.01^a^	3.08^a^
Fat content (%)	Blending	0	2.58^a^	2.56^a^	2.53^a^
		8	2.57^a^	2.59^a^	2.58^a^
	Fruit-on-the-bottom	0	2.63^a^	2.56^a^	2.57^a^
		8	2.59^a^	2.59^a^	2.61^a^
l-ascorbic acid content (mg/100 g)	Blending	0	8.2^a^	2.4^a^	1.1^a^
		8	3.1^b^	1.1^b^	0.2^b^
	Fruit-on-the-bottom	0	8.9^a^	2.6^a^	1.2^a^
		8	3.4^b^	1.7^b^	0.4^b^
Total phenolic content (mg GAE/100 g)	Blending	0	27.4^a^	32.9^a^	45.7^a^
		8	24.1^b^	27.6^b^	44.2^a^
	Fruit-on-the-bottom	0	28.1^a^	33.4^a^	47.8^a^
		8	25.0^b^	28.1^b^	45.9^a^
*Lactobacillus delbrueckii* counts (log cfu/g)	Blending	0	6.7^a^	6.8^a^	7.0^a^
		8	6.3^a^	6.5^a^	6.5^a^
	Fruit-on-the-bottom	0	6.5^a^	7.0^a^	6.9^a^
		8	6.2^a^	6.7^a^	6.9^a^
*Streptococcus thermophilus* counts (log cfu/g)	Blending	0	8.8^a^	8.7^a^	8.8^a^
		8	8.1^a^	8.4^a^	8.2^a^
	Fruit-on-the-bottom	0	8.7^a^	8.8^a^	8.7^a^
		8	8.7^a^	8.1^a^	8.6^a^

^a–c^Means in the same column followed by different lowercase represent significant difference (*p* < 0.05)

The increase in lactic acid during storage was higher in the yogurts containing fruit preparation on the bottom than for their blended counterparts for all fruits, which indicate that bacterial culture was metabolically more active in natural yogurt than in yogurt blended with fruit during storage. The fruit preparations used in our study did not contain any preservatives that may have caused lower viability of *S. thermophilus* and *Lb. delbrueckii* in the yogurts. Other factors such an acidification of the yogurt (by adding fruit preparation into the yogurt base), or the incorporation of ascorbic acid with fruit preparations, may be responsible for the difference in lactic acid contents between yogurts. The incorporation of ascorbic acid in yogurts can reduce the amount of oxygen required to the viability of *S. thermophilus* in blender yogurt (Talwalkar and Kailasapathy 2004).

The protein and fat content correspond to a typical fruit yogurt composition (Şengül et al. 2012). There were no significant differences in terms of total protein content between the fruit yogurts directly after the production and the yogurts stored 8 weeks. Probably, the change in the profile of nitrogen compounds occurs during the storage of yogurts, which cannot be observed by determining the total protein content using the Kjeldahl method. Serra et al. (2009) reported that the content of the hydrophobic peptides and free amino acids increased during storage of yogurt, which results from the hydrolysis of caseins and whey protein fractions. The total fat content of yogurt immediately after production did not differ significantly from that of yogurt after 8 weeks of storage. The Gerber method used in this work did not allow observation of oxidative changes in lipids during cold storage of yogurts. According to Citta et al. (2017) the presence of fruit puree in yogurt determines a protection against lipid peroxidation.

Strawberry and blueberry yogurts were shown to possess the highest L-ascorbic and total phenolic contents, respectively. There was no statistically significant difference in l-ascorbic acid and total phenolic content between blending and fruit-on-the-bottom yogurts. l-ascorbic acid concentration in yogurts was affected by storage time and decreased significantly. The content of phenolic compounds in the yogurt directly after the production was associated mainly with the amounts of the fruit preparations or the presence of small amount of phenolic compounds, which are derived from milk. Besle et al. (2010) reported that depending on the diet of cows, milk contained varying amount of ferulic acid and flavonoids. Similarly, the content of l-ascorbic acid in fruit yogurt mainly depended on the quantity of fruit preparation used. The concentration of vitamin C in milk is within the range 0.01–1.4 mg/100 mL, and it markedly is influenced by conditions of storage and heat treatment (Lindmark-Månsson and Åkesson 2000).

The total phenolic content in strawberry and sour cherry yogurts decreased significantly during 8 weeks of storage. In our study, the phenolic compounds from fruit preparation could interact with caseins or whey proteins causing the formation of soluble or insoluble complexes, which are responsible for the decrease of phenolic measurement.

As shown in Table 1, there was no significant difference in the counts of *Lactobacillus* and *S. thermophilus* among yogurts directly after the production and after 8 weeks of storage. The counts of *S. thermophilus* were higher than of *Lactobacillus* in all yogurts, which is consistent with studies carried out by Sun-Waterhouse et al. (2013) on yogurts with blackcurrant extract. During 8 weeks of storage, the viable counts of *Lactobacillus* species and *Streptococcus thermophilus* were above the recommended minimum limit of 6 log cfu/g. The viable counts of *S. thermophilus* and *Lactobacillus* species might influence the stability of anthocyanins during the storage of yogurts. The lactic acid bacteria can produce hydrogen peroxide and glycosidase enzymes, which can accelerate the destruction of anthocyanins during storage (Grimaldi et al. 2005; Jaroni and Brashears 2000).

### Anthocyanin content in fruit yogurts

Anthocyanin content of yogurts immediately after the production is shown in Tables 2, 3 and 4. The concentration of anthocyanins ranged from 6.70 mg/100 g in strawberry stirred yogurt to 24.61 mg/100 g in blueberry blending yogurt. The data on the amount of anthocyanin in fruit yogurt are scare. Anthocyanin content in strawberry yogurts in our study was different than the value reported by Oliveira et al. (2015) for strawberry yogurt. However, in this study, strawberry preparation was made using different ingredients, and the parameters of pasteurization were also different.

**Table 2:** Effect of storage and method of fruit preparation addition on anthocyanin content in strawberry yogurts

Peaks number	Anthocyanins	Type of yogurt	Storage time (weeks)					
			0	1	2	4	6	8
1	Cyanidin-3-glucoside	Blending	0.42^aA^	0.32^aB^	0.23^aC^	0.21^aCD^	0.18^aD^	0.16^aD^
		Fruit-on-the-bottom	0.45^aA^	0.36^aB^	0.29^bC^	0.26^bCD^	0.24^bD^	0.23^bD^
2	Pelargonidin-3-glucoside	Blending	5.29^aA^	3.85^aB^	3.04^aC^	2.52^aD^	2.13^aDE^	1.92^aE^
		Fruit-on-the-bottom	5.34^aA^	4.20^bB^	3.56^bC^	3.20^bD^	2.93^bDE^	2.77^bE^
3	Pelargonidin-3-rutinoside	Blending	0.44^aA^	0.32^aB^	0.23^aC^	0.18^aD^	0.17^aD^	0.16^aD^
		Fruit-on-the-bottom	0.42^aA^	0.33^aB^	0.27^bC^	0.26^bC^	0.25^bDC^	0.23^bD^
4	Pelargonidin 3-malonyl-glucoside	Blending	0.55^aA^	0.40^aB^	0.33^aC^	0.32^aC^	0.24^aD^	0.21^aD^
		Fruit-on-the-bottom	0.56^aA^	0.41^aB^	0.36^aC^	0.34^aC^	0.32^bCD^	0.30^bD^
	Total	Blending	6.70^aA^	4.89^aB^	3.83^aC^	3.23^aD^	2.73^aDE^	2.45^aE^
		Fruit-on-the-bottom	6.77^aA^	5.30^aB^	4.48^bC^	4.07^bCD^	3.75^bDE^	3.53^bE^

^a–b^Means in the same column followed by different lowercase represent significant difference (*p* < 0.05)

^A–E^Means in the same line followed by different uppercase represent significant difference (*p* < 0.05)

**Table 3:** Effect of storage and method of fruit preparation addition on anthocyanin content of sour cherry yogurts

Peaks number	Anthocyanins	Type of yogurt	Storage time (weeks)					
			0	1	2	4	6	8
5	Cyanidin-3-sophoroside	Blending	0.81^aA^	0.60^aB^	0.47^aC^	0.41^aD^	0.37^aDE^	0.34^aE^
		Fruit-on-the-bottom	0.76^aA^	0.62^aB^	0.56^bB^	0.52^bBC^	0.50^bBC^	0.46^bC^
6	Cyanidin-3-glucosyl-rutinoside	Blending	6.60^aA^	5.14^aB^	4.09^aC^	3.48^aD^	3.13^aDE^	2.87^aE^
		Fruit-on-the-bottom	6.76^aA^	5.41^bB^	4.82^bC^	4.45^bD^	4.18^bE^	4.05^bE^
7	Cyanidin-3-xylorutinoside	Blending	0.09^aA^	0.08^aA^	0.07^aA^	0.06^aAB^	0.05^aB^	0.04^aB^
		Fruit-on-the-bottom	0.10^aA^	0.10^aA^	0.07^aB^	0.07^aB^	0.07^aB^	0.06^bB^
1	Cyanidin-3-glucoside	Blending	0.48^aA^	0.35^aB^	0.30^aBC^	0.26^aCD^	0.23^aCD^	0.22^aD^
		Fruit-on-the-bottom	0.44^aA^	0.39^aAB^	0.34^aB^	0.33^aB^	0.31^bB^	0.27^bC^
8	Cyanidin-3-rutinoside	Blending	3.04^aA^	2.20^aB^	1.85^aC^	1.58^aCD^	1.41^aD^	1.34^aD^
		Fruit-on-the-bottom	2.98^aA^	2.48^bB^	2.17^bBC^	1.91^bC^	1.80^bC^	1.80^bC^
9	Petunidin-3-rutinoside	Blending	0.09^aA^	0.08^aA^	0.06^aB^	0.05^aB^	0.05^aB^	0.04^aB^
		Fruit-on-the-bottom	0.09^aA^	0.08^aA^	0.06^aB^	0.05^aB^	0.05^aB^	0.05^aB^
	Total	Blending	11.11^aA^	8.45^aB^	6.84^aC^	5.84^aD^	5.24^aDE^	4.85^aE^
		Fruit-on-the-bottom	11.13^aA^	9.08^aB^	7.87^bC^	7.33^bCD^	6.91^bD^	6.69^bD^

^a–b^Means in the same column followed by different lowercase represent significant difference (*p* < 0.05)

^A–E^Means in the same line followed by different uppercase represent significant difference (*p* < 0.05)

**Table 4:** Effect of storage and method of fruit preparation addition on anthocyanin content of blueberry yogurts

Peaks number	Anthocyanins (M^+^ m/z)	Type of yogurt	Storage time (weeks)					
			0	1	2	4	6	8
20	Malvidin-3-galactoside (493)	Blending	4.11^aA^	3.72^aB^	3.40^aC^	3.35^aCD^	3.22^aD^	3.14^aD^
		Fruit-on-the-bottom	4.04^aA^	3.71^aB^	3.57^aBC^	3.47^bBC^	3.40^bCD^	3.37^bD^
21	Malvidin-3-glucoside (493)	Blending	5.68^aA^	5.01^aB^	4.82^aBC^	4.67^aC^	4.54^aC^	4.40^aC^
		Fruit-on-the-bottom	5.59^aA^	5.30^bB^	5.16^bBC^	5.00^bBC^	4.88^bC^	4.70^bC^
22	Malvidin-3-arabinoside (463)	Blending	1.48^aA^	1.35^aB^	1.30^aBC^	1.25^aC^	1.17^aC^	1.12^aC^
		Fruit-on-the-bottom	1.49^aA^	1.39^aB^	1.32^aBC^	1.28^bC^	1.23^bC^	1.22^bC^
10	Delphinidin-3-galactoside (465)	Blending	0.84^aA^	0.72^aB^	0.65^aBC^	0.59^aCD^	0.56^aD^	0.56^aD^
		Fruit-on-the-bottom	0.84^aA^	0.70^aB^	0.65^aBC^	0.64^aCD^	0.62^bD^	0.62^bD^
11	Delphinidin-3-glucoside (465)	Blending	1.34^aA^	1.11^aB^	1.01^aBC^	0.98^aC^	0.97^aC^	0.93^aC^
		Fruit-on-the-bottom	1.27^aA^	1.13^aB^	1.06^aBC^	0.98^aC^	0.95^aC^	0.95^aC^
13	Delphinidin-3-arabinoside (435)	Blending	0.78^aA^	0.67^aB^	0.61^aBC^	0.54^aC^	0.53^aC^	0.52^aC^
		Fruit-on-the-bottom	0.85^aA^	0.74^aB^	0.68^aBC^	0.65^aBC^	0.63^aC^	0.62^aC^
14	Petunidin-3-galactoside (479)	Blending	0.90^aA^	0.72^aB^	0.65^aBC^	0.61^aC^	0.59^aC^	0.58^aC^
		Fruit-on-the-bottom	0.90^aA^	0.82^bAB^	0.77^bB^	0.73^bBC^	0.71^bCD^	0.66^bD^
16	Petunidin-3-glucoside (479)	Blending	1.73^aA^	1.46^aB^	1.33^aBC^	1.25^aC^	1.25^aC^	1.22^aC^
		Fruit-on-the-bottom	1.74^A^	1.60^bAB^	1.50^bBC^	1.40^bC^	1.35^bC^	1.31^bC^
19	Petunidin-3-arabinoside (449)	Blending	0.44^aA^	0.40^aAB^	0.36^aBC^	0.34^aC^	0.32^aC^	0.31^aC^
		Fruit-on-the-bottom	0.41^aA^	0.36^aAB^	0.36^aB^	0.34^aB^	0.32^bB^	0.30^bB^
12	Cyanidin derivatives*	Blending	1.24^aA^	1.03^aB^	0.93^aC^	0.93^aC^	0.90^aC^	0.88^aC^
1		Fruit-on-the-bottom	1.32^aA^	1.18^bB^	1.09^bBC^	1.07^bCD^	1.06^bD^	1.98^bD^
15								
17	Peonidin derivatives^+^	Blending	0.31^aA^	0.25^aB^	0.23^aB^	0.22^aB^	0.22^aB^	0.22^aB^
18		Fruit-on-the-bottom	0.36^aA^	0.33^bAB^	0.30^bB^	0.29^bB^	0.28^bB^	0.26^bB^
23–30	Acylated anthocyanins^#^	Blending	5.76^aA^	5.35^aB^	5.02^aC^	4.84^aCD^	4.69^aDE^	4.51^aE^
		Fruit-on-the-bottom	5.59^aA^	5.41^aB^	5.18^bC^	4.99^bD^	4.90^bDE^	4.77^bE^
	Total	Blending	24.61^aA^	21.77^aB^	20.31^aC^	19.57^aD^	18.97^aDE^	18.38^aE^
		Fruit-on-the-bottom	24.40^aA^	22.66^aB^	21.64^bC^	20.84^bD^	20.34^bDE^	19.78^bE^

*Cyanidin derivatives: cyanidin-3-galactoside (M^+^ m/z 449; peak number—12), cyanidin-3-glucoside (M^+^ m/z 449; peak number—1), cyanidin-3-arabinose (M^+^ m/z 419; peak number—15), ^+^ peonidin derivatives: peonidin-3-galactoside (M^+^ m/z 463; peak number—17), peonidin-3-glucoside (M^+^ m/z 463; peak numer—18),#—acylated anthocyanins (M^+^ m/z 463, 501, 505, 521, 531, 535, 573/505, 574/535; peaks number 23, 26, 28, 27, 24, 25, 29, 30, respectively)

^a–b^Means in the same column followed by different lowercase represent significant difference (*p* < 0.05)

^A–E^Means in the same line followed by different uppercase represent significant difference (*p* < 0.05)

**Fig. 1:**
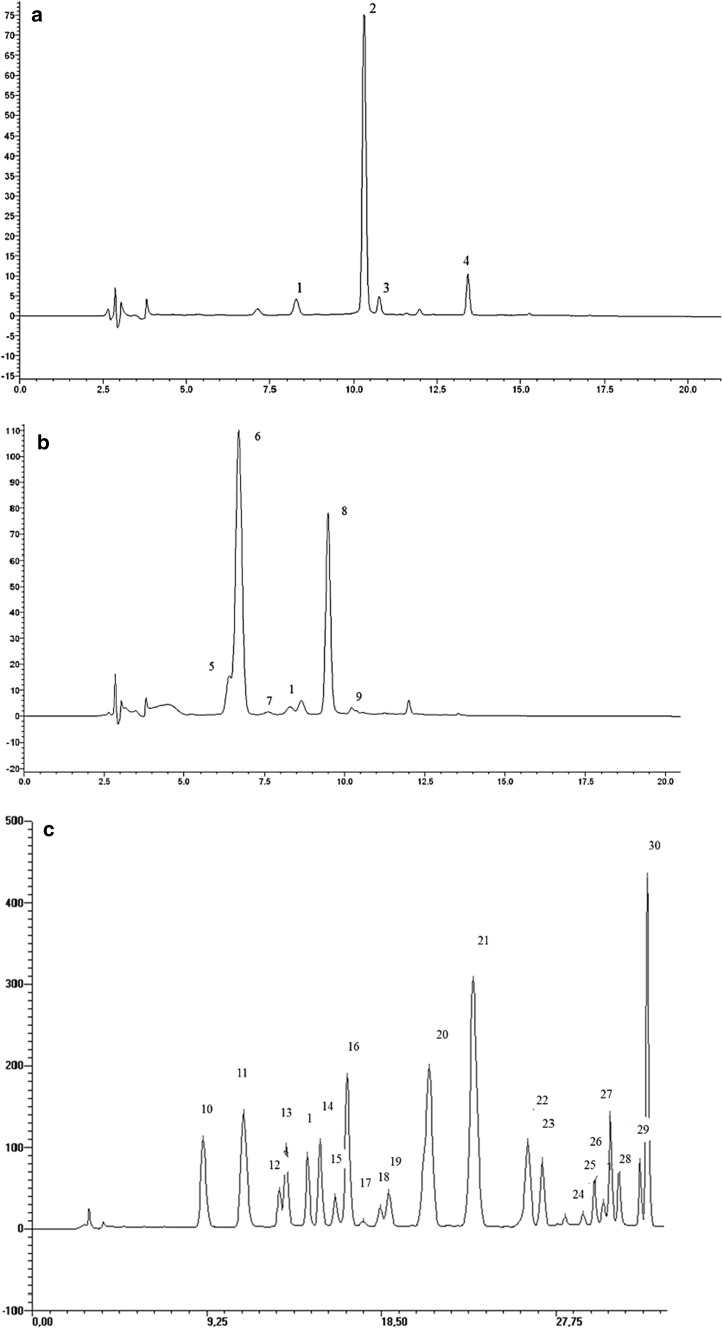
HPLC chromatograms of anthocyanins in strawberry (**a**), sour cherry (**b**) and blueberry (**c**) yogurts. The numbers above the peaks refer to Table 2 (**a**), Table 3 (**b**) and Table 4 (**c**)

The predominant anthocyanin present in strawberry yogurts was pelargonidin-3-glucoside, which constitutes about 79% of the total anthocyanin content (Fig. 1a). The second most abundant anthocyanin in strawberry yogurts was pelargonidin-*3*-malonyl-glucoside. Small amounts of cyanidin-3-glucoside and pelargonidin-3-rutinoside were also present. The HPLC profile of anthocyanins in the strawberry samples was in agreement with previous findings (García-Viguera et al. 1999).

Cyanidin-3-glucosyl-rutinoside was the predominant compound in cherry yogurts followed by cyanidin-3-rutinoside and cyanidin-3-sophoroside, representing 27% and 7% of total anthocyanins, respectively (Fig. 1b). Petunidin-3-rutinoside and cyanidin-3-xylorutinoside were present only in small amount. The result is in agreement with earlier studies indicating that cyanidin was the major anthocyanidin in the cherry nectars and jams (Ertan et al. 2018; Picariello et al. 2017).

Compared with other fruits, blueberries have a complex mixture of anthocyanins (Fig. 1c). The blueberry yogurts contained primarily malvidin derivatives followed by equal amounts of petunidin and delphinidin derivatives as well as very low amounts of cyanidin and peonidin glycosides. Derivatives of malvidin accounted for 46% of the total anthocyanin. Moreover, glucosides were found to be present in much greater quantity than galactosides and arabinosides. Giovanelli and Buratti (2009) have reported the content of monomeric anthocyanins in fruit of Patriot cultivar grown in Italy. Compared to our data, the fresh fruit contained relatively higher levels of delphinidin derivatives and lower levels of glucosides than blueberry used in our study. The variation could be related to the harvest date, temperature of storage and geographical origin of the blueberry fruit. Mallik and Hamilton (2017) showed that late harvest and low temperature storage significantly increased anthocyanin content for wild blueberries. Moreover, according to Ichiyanagi et al. (2001), high temperature during the production of blueberry preparation may affect the composition of anthocyanin. It was observed that during heating the arabinosides were more unstable than glucosides and galactosides, when the glycosides with the same aglycon were compared. In our study, similar hydrolysis could take place during blueberry preparation production, particularly during pasteurization step.

### Change the anthocyanin content during storage of yogurts

Anthocyanin content in yogurts showed a significant decrease during storage, especially for the first 2 weeks (Tables 2, 3, 4). Anthocyanin content was more affected by the storage time than by the method of addition of fruit preparation on yogurt. The losses of anthocyanins compared to the freshly produced yogurt were about 11–43% after 2 weeks and 19–63% after 8 weeks of storage. Linear regression analysis confirmed that degradation of anthocyanins in yogurt followed first-order reaction kinetics. These results are in agreement with the previous studies reporting first-order reaction kinetics for anthocyanins present in yogurt with purple carrot and Peruvian berry (Wallace and Giusti 2008). Kinetic parameters included first-order reaction rate constants (*k*) and half-life (*T*_1/2_—time needed for the degradation of 50% of initial anthocyanin contents under the experimental conditions) are shown in Table 5. The half-life of anthocyanin in fruit-on-the-bottom yogurts was 39–63% higher than the *T*_1/2_ of the blending samples and, consequently, the rate of degradation of the blending samples was higher. Our results are comparable to these obtained by Oliveira et al. (2015), who reported that the rate of pelargonidin-3-glucoside degradation in strawberry preparation was markedly affected by yogurt addition. He et al. (2016) suggested that the weak interaction of non-covalent nature between protein and anthocyanins could improve the stability of pigment. This observation does not find the confirmation in our data, which could be because our yogurt was made from milk, which has been ultra-high temperature pasteurized (UHT). Due to the high temperature whey proteins were denatured and coagulated (Oliveira et al. 2015), which could affect the interaction of anthocyanins and proteins in our yogurts.

**Table 5:** The kinetic parameters for the degradation of total anthocyanins in fruit yogurts during storage

Fruit preparation	Type of yoghurt	-k^a^ (weeks^−1^)	T_1/2_ (weeks)
Strawberry	Blending	0.126	5.5
	Fruit-on-the-bottom	0.081	8.5
Sour cherry	Blending	0.104	6.7
	Fruit-on-the-bottom	0.063	10.9
Blueberry	Blending	0.036	19.0
	Fruit-on-the-bottom	0.026	26.4

^a^Rate of degradation

Significantly higher loss of anthocyanins in blended yogurt was also possible due to higher concentration of oxygen absorbed during the stirring process promoting the degradation of anthocyanins. Moreover, the difference can be related to the greater anthocyanin degradation at higher pH (Wallace and Giusti 2008). Since the pH of both types of yogurts after mixing was similar, this difference could be attributed to the fact that the pH of fruit preparation was much lower. The pH of yogurts ranged from 3.94 to 4.15, but the pH of fruit preparations was 3.18 ± 0.24 for strawberry, 2.92 ± 0.18 for sour cherry, and 3.05 ± 0.30 for blueberry, respectively (data not shown). The slower rate of pigment degradation in fruit-on-the-bottom yogurts can be attributed to the fact that anthocyanins were present in the fruit preparation but in blending yogurts anthocyanins were present in the whole product, the pH of which was significantly higher than the fruit preparation.

Other possible causes for the observed faster rate of anthocyanin degradation in blending yogurt is that the lactic acid bacteria can produce hydrogen peroxide, which not only can inhibit the growth of undesirable organism, but also would accelerate the destruction of anthocyanins. Because lactic acid bacteria are catalase-negative, hydrogen peroxide can accumulate to high levels in the growth medium (Jaroni and Brashears 2000). It is possible that anthocyanins contained in the fruit preparation on fruit-on-the-bottom yogurts have been less exposed to hydrogen peroxide than the pigments present in the whole yogurt.

Hydrolysis of glycosidic substituents by glycosidase enzymes of microbial origin can also result in pigment destruction. Grimaldi et al. (2005) showed that the *Lactobacillus* and *Pediococcus* species had α- and β-d-glucopyranosidase (β-d-glucosidase) activities, but the small amount of fructose and glucose in the solution (0.1–20 g/L) has been shown to be inhibitory to these enzymes. In our study, glucose and fructose content in yogurts was at a high level, and it resulted from the natural content in fruit and from the hydrolysis of sucrose, which could occur during the production of fruit preparation. The high level of reducing sugars in yogurt could reduce the activity of β-d-glucosidase because similar changes in individual anthocyanin compounds (regardless of the attached sugar) present in fruit yogurt were found. Moreover, although the aglycon was suggested to be formed in the stored yogurt, we could not detect any additional peak formation in the chromatograms.

Comparing the rate of anthocyanins degradation during storage yogurt obtained from different species of fruit, it was found that there are considerable differences (Table 5). The highest stability has anthocyanins contained in blueberry yogurts. The half-life of the pigments in blender yogurts for strawberry, sour cherry, and blueberry was found to be 5.5, 6.7, and 19.0 weeks, respectively. The differences in anthocyanin structure can affect pigment stability. The presence of acylated anthocyanin in the blueberry as compared to nonacylated pigments in strawberry and sour cherry might be responsible for its enhanced stability. Furthermore, differences in main aglycon of different fruit (malvidin in blueberry vs. pelargonidin and cyanidin in strawberry and sour cherry) might account for the improved stability showed by blueberry anthocyanins. The content of other components in yogurt matrix may also be important in the observed lower stability of anthocyanin in strawberry products. The strawberry yogurt contains a higher level of ascorbic acid than yogurt with sour cherry and blueberry fruits (Table 1). The presence of ascorbic acid in strawberry preparation could accelerate anthocyanins decay during storage of yogurt. Hydrogen peroxide or furfural formed during oxidative degradation of ascorbic acid could interact with anthocyanins and accelerate the degradation rate (Cao et al. 2012). The high stability of pigment in blueberry yogurt could be associated with the presence of acylated anthocyanins in this fruit. Wallace and Giusti (2008) reported a protective effect of fat from yogurt on acylated anthocyanins from purple carrot, but this effect was not observed on yogurt sample with fruit containing only non-acylated pigments. Similarly, in our study, the presence of fat in yogurt could stabilize acylated anthocyanins in the blueberries yogurt, but does not affect the non-acylated pigment in strawberry and sour cherry yogurt.

### Changes in anthocyanin profile during storage of fruit yogurt

Similar changes were found in individual anthocyanin compounds during storage of strawberry yogurt, depending on the production method. During storage of blending yogurt the content of pelargonidin-3-glucoside and pelargonidin-3-malonyl-glucoside was reduced by 64% and 63% of the initial value, respectively. The content of other anthocyanins decreased about 62–63%. For fruit-on-the-bottom yogurt, the level of losses of pelargonidin-3-glucoside and pelargonidin-3-malonyl-glucoside after 8 weeks of storage were 48% and 47%, respectively. This is in accordance with García-Viguera et al. (1999) who reported no difference in the stability of individual anthocyanin during storage of strawberry jams. Similar results were obtained for yogurt with sour cherry. After 8 weeks of storage, the concentration of individual anthocyanins in blending and fruit-on-the-bottom yogurts decreased by 54–58% and 39–44%, respectively.

Anthocyanin degradation during storage of blueberry yogurt depended not only on the method of fruit preparation addition but also on the molecular structure of anthocyanins. A significant alteration in the pigment profile was observed. The proportion of malvidin-glycosides and acylated anthocyanins increased with time of storage, with a concomitant decrease in the proportion of petunidin, delphinidin, and peonidin derivatives. The percentage of loss of malvidin derivatives during storage of blending yogurt was 21–24%, while the loss of other non-acylated anthocyanin ranged from 30 to 34%. Furthermore, there was less than 22% loss of acylated anthocyanins during storage of blending yogurt. Similar observation was made for yogurt with blueberry preparation on the bottom of the package. This finding is in accordance with earlier studies that have found that the malvidin-glycosides and acylated anthocyanins show high stability during processing and storage of blueberry products. This may be attributed to the chemical structure of these pigments. The malvidin-glycosides stability is due to the two methoxy substituents adjacent in the 3′ and 5′ positions on the B-ring, which makes it the least reactive of the all monomeric anthocyanins (Skrede et al. 2000). Likewise, the acylation of the sugar moiety in anthocyanins with aromatic acids decreases polarity and confers higher stability during processing and storage than other natural pigments. Anthocyanins with acylating substituents also show remarkable stability in the pH of dairy products (Giusti and Wrolstad 2003, Wallace and Giusti 2008).

### Color changes during storage of fruit yogurt

The color of fruit yogurt has a remarkable influence on consumer acceptance and is also an indicator of the changes in pigment concentration that occur during storage. Table 6 shows the changes in instrumental color parameters for blending and fruit-on-the-bottom yogurt samples. A general increase in L* value was observed during the storage period, although blender yogurt showed a faster increase than those with fruit-on-the-bottom products. However, no significant differences were noticed when analyzing the L* value in blending and fruit-on-the-bottom blueberry yogurts. The degradation of anthocyanins might have resulted in the significant changes in brightness during storage of yogurt. These results were in accordance with those found by Hassani and Sharifi (2012) who reported that the lightness of barberry yogurt increased during storage.

**Table 6:** Effect of storage and method of fruit preparation addition on color parameters (L*, h°, C*) of fruit yogurts

Parameters	Storage time (weeks)	Strawberry yogurt		Sour cherry yogurt		Blueberry yogurt	
		Blending	Fruit-on-the-bottom	Blending	Fruit-on-the-bottom	Blending	Fruit-on-the-bottom
Lightness (L*)	0	72.3^dA^	71.8^bA^	66.7^cA^	66.8^bA^	55.6^bA^	55.5^aA^
	1	74.1^cdA^	73.2^aA^	67.7^bA^	67.3^abA^	56.0^abA^	55.5^aA^
	2	75.1b^cA^	73.9^aB^	68.2^abA^	67.5^abA^	56.3^aA^	55.7^aA^
	4	75.7^abA^	74.2^aB^	68.6^aA^	67.7^aB^	56.5^aA^	55.8^aA^
	6	76.0^aA^	74.3^aB^	68.7^aA^	67.8^aB^	56.6^aA^	55.9^aA^
	8	76.3^aA^	74.4^aB^	68.8^aA^	67.8^aB^	56.7^aA^	55.9^aA^
Hue angle (h°)	0	21.5^eA^	21.8^dA^	1.7^dA^	1.8^bA^	329.3^aA^	329.9^aA^
	1	26.2^dA^	23.6^cB^	2.1^cA^	2.0^abA^	329.0^aA^	329.8^aA^
	2	29.7^cA^	24.9^bcB^	2.3^bcA^	2.2^aA^	328.6^aA^	329.8^aA^
	4	32.4^bA^	25.9^abB^	2.5^abA^	2.2^aA^	328.4^aA^	329.8^aA^
	6	34.3^abA^	26.6^aB^	2.6^aA^	2.3^aA^	328.3^aA^	329.9^aA^
	8	35.3^aA^	26.9^aB^	2.7^aA^	2.3^aB^	328.3^aA^	329.9^aA^
Chroma (C*)	0	10.2^aA^	10.2^aA^	15.5^aA^	15.5^aA^	18.0^aA^	17.9^aA^
	1	9.9^aA^	9.9^aA^	14.2^bA^	14.7^bA^	16.5^bA^	16.9^bA^
	2	9.7^aA^	9.7^aA^	13.4^cB^	14.3^cA^	15.6^cB^	16.5^cA^
	4	9.6^aA^	9.6^aA^	12.9^cdB^	14.1^cA^	15.0 ^dB^	16.2^cdA^
	6	9.6^aA^	9.6^aA^	12.6^cdB^	14.0^cA^	14.7^deB^	16.1^dA^
	8	9.6^aA^	9.6^aA^	12.4 ^dB^	14.0^cA^	14.5^eB^	16.0^dA^

^a–e^Means in the same column followed by different lowercase represent significant difference (*p* < 0.05)

^A–B^Means in the same line followed by different uppercase represent significant difference (*p* < 0.05)

Strawberry and sour cherry yogurts showed the loss of redness during storage as evidenced by change in hue angle. Yogurt samples demonstrated a significant increase of this parameter, indicating that the color was changing from red toward orange during storage, most probably due to degradation of anthocyanins or/and the formation of yellow and brown polymerization compounds. This change was more pronounced in strawberry samples. On the contrary, the degree of hue angle remained almost constant during storage of blueberry yogurts. There was a significant effect of method of fruit preparation addition on h° value for strawberry and sour cherry products. After 1 week of storage, the blending strawberry yogurt had a significantly higher h° value than the fruit-on-the-bottom yogurt. The same was observed for the sour cherry yogurts, but after 8 weeks. However, blending and fruit-on-the-bottom blueberry yogurts presented the same hue angle, which remained almost constant until the end of the storage.

During storage, chroma (C*) decreased slightly in all products, indicating that the color of yogurt became less intense with time. The impact of the production method on the chroma of yogurt was significant for sour cherry and blueberry yogurt. After 2 weeks, the blending sour cherry and blueberry yogurts had a significantly lower chroma than the fruit-on-the-bottom yogurts. No significant differences were noticed for strawberry yogurts.

Differences in the color parameters of blending and fruit-on-the-bottom yogurts can be attributed to different content of the anthocyanins. Nevertheless, the differences found in color between yogurts appear less significant than found in pigment concentration, owing to the fact that the presence of other compounds in fruit yogurt also had a considerable effect on the color. Fruit yogurts contain high concentrations of phenolic compounds (Table 1) such as phenolic acids and flavan-3-ols, which are known to interact with anthocyanins and greatly enhance the color intensity (Pascual-Teresa and Sanchez-Ballesta 2008; Patras et al. 2010).

## Conclusion

The results clearly indicate the role of method of addition of fruit preparation on the anthocyanin concentration of fruit yogurts. The addition of fruit preparation on bottom of package compared to mixed products increased the half-life time of anthocyanins by 3, 4.2, 7.4 weeks in strawberry, sour cherry, and blueberry yogurts, respectively. These suggest that the separation of yogurt from fruit preparation could make a considerable importance from a nutritional standpoint. In food technology practice, these conclusions have also important implications for fruit yogurt producers, to develop an effective strategy for the production of yogurt with high concentrations of desirable fruit antioxidants. The level of losses in the content of anthocyanin during storage of yogurt also depends on the fruit species. The degradation rate was more pronounced in products with strawberry and sour cherry than with blueberry fruit. Therefore, the addition of colorants in the production of blueberry yogurts is not recommended because the natural pigments contained in blueberry are stable and give an intense color to yogurt during the entire storage period.
